# The Population Growth of *Spodoptera frugiperda* on Six Cash Crop Species and Implications for Its Occurrence and Damage Potential in China

**DOI:** 10.3390/insects11090639

**Published:** 2020-09-17

**Authors:** Wenwen Wang, Pengyang He, Yiyang Zhang, Tongxian Liu, Xiangfeng Jing, Shize Zhang

**Affiliations:** State Key Laboratory of Crop Stress Biology for Arid Areas, Northwest A&F University, Yangling 712100, China; wangwenwen@nwafu.edu.cn (W.W.); hepengyang@nwafu.edu.cn (P.H.); zhangyiyang@nwafu.edu.cn (Y.Z.); txliu@nwafu.edu.cn (T.L.)

**Keywords:** *Spodoptera frugiperda*, invasive pest, life history, population growth, damage potential, cash crop

## Abstract

**Simple Summary:**

The fall armyworm *Spodoptera frugiperda* is an invasive pest, which can cause severe economic losses by larvae feeding on a variety of crops. To develop effective control technology, it is particularly necessary to study the basic biology and ecology of this invasive insect. This experiment investigated the development, survival, and reproduction and population growth of *S. frugiperda* on six cash crop species. This study indicated that *S. frugiperda* fed on maize and wheat had shorter preadult developmental durations, higher preadult survival, greater pupal weights and higher fecundity compared to the other four plants. Moreover, although the young larvae of *S. frugiperda* feeding on Chinese cabbage had a high mortality rate, the old larvae were voracious, which might still cause economic losses to Chinese cabbage. Our results showed that *S. frugiperda* could cause great economic losses to these cash crops, which should attract the attention of agricultural management departments.

**Abstract:**

*Spodoptera frugiperda* is a significant migratory invasive pest, identified as a serious threat to agricultural production and food security in China. However, to our knowledge, the effects of most host plants on the biological characteristics of *S. frugiperda* have not been well studied. To develop effective management strategies for *S. frugiperda* in its new invasive habitat, basic biological and ecological knowledge of this pest are crucial requirements. Here, we examined the effects of six cash crops maize, wheat, soybean, tomato, cotton and Chinese cabbage on the development, survival, fecundity of *S. frugiperda* by using the age-stage, two-sex life table. The preadult stage, adult preoviposition period and total preoviposition period of *S. frugiperda* were shortest on maize and wheat but were longest on tomato. Fecundity was greatest on maize and wheat but smallest on tomato. The highest intrinsic rate of increase, finite rate of increase, net reproductive rate and the shortest mean generation time were recorded on maize. This present study showed that *S. frugiperda* could cause great economic losses to these cash crops, which should attract the attention of agricultural management departments. Our findings provide useful information in predicting population dynamics and understanding the potential damage that could be incurred by *S. frugiperda* invasion.

## 1. Introduction

The fall armyworm, *Spodoptera frugiperda* (J.E. Smith) (Lepidoptera: Noctuidae), which originated in the tropical and subtropical regions of America, has been identified as a notorious polyphagous pest with high migration ability, a wide range of hosts, voracious larval feeding and high fecundity; this pest is known to cause heavy economic damage to crops and pastures worldwide [1,2,3]. *Spodoptera frugiperda* consists of two haplotypes: corn strain and rice strain. The corn-strain haplotype mainly feeds on corn, cotton and sorghum, while the rice-strain haplotype invades rice and pastures [4].

In Asia, *S. frugiperda* was first detected in India in 2018 and later in other countries, which include Myanmar, Thailand, Yemen and Sri Lanka [5,6]. In January 2019, the invasion of *S. frugiperda* was confirmed for the first time in Yunnan Province, China [7]. By October 2019, it has already spread to 26 Chinese provinces [8]. In China, *S. frugiperda* could possibly move northward via seasonal monsoons in spring and summer to enter wheat, corn and other major crop production areas along the Yangtze River Basin, the Yellow River Basin and the northeast region of China; therefore, it is considered as a serious threat to Chinese agricultural production and food security [9]. At present, management of *S. frugiperda* primarily depends on broad spectrum chemical insecticide applications, which are noxious to beneficial arthropods [10,11]. Although insecticidal control remains a primary tactic for managing *S. frugiperda*, extensive use of insecticides has led to the development of resistance of this pest to conventional insecticidal compounds and *Bacillus thuringiensis* toxins [12,13]. Thus, it is a must to develop sustainable control methods to manage this insect.

To develop effective management strategies for *S. frugiperda* in its new invasive habitat, basic biological and ecological knowledge of this pest are crucial requirements. Reportedly, *S. frugiperda* has the potential to damage 353 species of plants belonging to 76 plant families [2]. However, to our knowledge, the effects of most host plants on the biological characteristics of *S. frugiperda* have not been well studied. In China, maize has been identified as the staple food crop, which is widely distributed throughout the country. Generally speaking, a spring maize area is distributed in east and north of China, a summer maize area in the Huanghuaihai Plain, an irrigation maize area in northwest China, a mountain maize area in southwest China and a hilly maize area in south China and the Qinghai-Tibet plateau [14,15]. Additionally, wheat, cotton, soybean and vegetables, including maize, are also widely cultivated in these areas. Moreover, the planting seasons of these crops are often overlapping or continuous in different regions of China, which could provide sufficient food resources for the occurrence and migration of *S. frugiperda.* It is well known that plant species significantly affect the survival, fecundity and population growth of herbivorous insects [16]. The impact of plant species that slow or accelerate herbivore development should be taken into account when designing and developing integrated pest managements. Therefore, investigating the effects of these crops on the growth, development, survival, reproduction and population dynamics of *S. frugiperda* is of great significance to make a comprehensive control strategy and predict the occurrence of the population.

The cohort life table gives the most comprehensive description of the survivorship, development and reproduction of a population, so it is a fundamental to both theoretical and applied population ecology. However, the traditional female and age-specific life tables ignore both the male component of a population and stage differentiation, thereby limiting their practical application [17,18,19]. To comprehensively understand and devise practical applications for *S. frugiperda* demography, it is necessary to collect demographic data based on the age-stage, two-sex life table when the pest feeds on different crop species.

In this study, we collected life table data for *S. frugiperda* individuals reared on six host plant species and compared their demographic characteristics using the age-stage, two-sex life table. Finally, the population growth of *S. frugiperda* cohorts reared on different plants was projected using a computer simulation. This current study provides comprehensive insight into the population growth and damage potential of *S. frugiperda* and can also be used for developing targeted strategies for fall armyworm prevention.

## 2. Materials and Methods

### 2.1. Insect Culture

In July 2019, *S. frugiperda* egg masses were collected from a maize field in Yangling, Shaanxi, China. All egg masses were incubated at room temperature, and approximately 50 newly hatched neonate larvae were reared on the same plant species in nylon mesh-covered cages (60 × 60 × 60 cm) in an artificial climate room. The conditions in the climate room were as follows: 25–30 °C, 50–80% RH and a 16/8 h (light/dark, L/D) photoperiod. The insects were reared on respective host plants for at least one generation in the climate room.

### 2.2. Plant Species

Six plant species were used in this study: maize (*Zea mays* L. var. Tiancheng 288), wheat (*Triticum aestivum* L. var. Xi’nong 979), soybean [*Glycine max* (L.) Merr. var. Zhonghuang 37], tomato (*Solanum lycopersicum* L. var. Maofen 802), cotton (*Gossypium hirsutum* L. var. Luzao) and Chinese cabbage [*Brassica pekinensis* (Lour.) Rupr. var. Qinza 2]. Plant seeds were purchased from Yangling Nongcheng Seed Supplement Company (Yangling, China). All plant seeds were individually sown in plastic pots (10 × 15 cm), which contained a 3:1:1 mixture of commercial peat moss (Pindstrup Mosebrug A/S, Ryomgaard, Denmark), perlite and vermiculite. All plants were kept in the same climate room. At the beginning of the experiments, the maize, wheat, soybean and cotton plants were all 14 days old; the tomato and Chinese cabbage plants were four weeks old.

### 2.3. Life Table Study of S. frugiperda

Six *S. frugiperda* egg masses (about 200 eggs) laid within 6 h were randomly collected from the climate room, within the same colony, and kept in a Petri dish (Haimen Zhongtai Experimental Equipment Company, Haimen, China) (12.0 cm in diameter and 2.0 cm in height) until hatching started. A piece of filter paper was placed at the bottom of the Petri dish, and a few drops of water were added as needed to maintain high humidity (approximately 60–70% RH). The eggs were inspected carefully every 6 h, and the number of larvae that hatched was also recorded. The single first instar was respectively transferred from the Petri dishes to a 25 mL plastic cup (3 × 4 × 3.5 cm) with small holes pierced through the lid using a soft camel hairbrush. The total numbers of first instar larvae used were 113, 101, 101, 103, 104, and 112 larvae for maize, wheat, soybean, tomato, cotton and Chinese cabbage, respectively. The leaves were replaced every 24 h to avoid microbial contamination. The surviving larvae were checked every day. The newly pupated larvae were collected every 24 h and individually weighed using an electronic balance (Mettler-Toledo XS64, Greifensee, Switzerland), with a precision of 0.1 mg. After weighing, the pupae were sexed, and then placed in a plastic cup lined with cotton, respectively. Each plastic cup had one pupa, which was covered with a black plastic bag to block the light. The pupae were checked daily until adult emergence. The newly emerged adults from the same plant species were paired and kept in individual transparent plastic cylindrical boxes (8.5 × 6 cm). Totally, there were 25, 19, 21, 24 and 39 pairs for maize, wheat, soybean, tomato and cotton, respectively. A cotton ball soaked with 10% sucrose water was used to supply nutrition, and a piece of creased buffer paper was supplemented as an oviposition substrate. The buffer paper and cotton ball were replaced once a day until the adults died. If the male died, a new male from the same mass-reared colony was supplemented until the female died. Newly laid egg masses were transferred to a new box, and the total number of eggs was recorded. All experiments were conducted in climatic chambers at 25 ± 1 °C, 60–70% RH and a 16/8 h (light/dark, L/D) photoperiod.

### 2.4. Life Table Data Analysis

The raw life table data of individual *S. frugiperda* were analysed using the TWO-SEX-MSChart program [20], based on the age-stage, two-sex life table theory [21] and the method described by Chi [22]. The survival rate (*s_xj_*) (*x* = age, *j* = stage), which is the probability that a newly laid egg will survive to age *x* and stage *j*, and fecundity *f_xj_*, which is the number of hatched eggs produced by an adult female at age *x*, were calculated.

Age-specific survival rate (*l_x_*) was calculated as

(1) $$l_{x}=\overset{m}{\underset{j=1}{\sum }}s_{xj}$$

where *m* is the number of stages.

Age-specific fecundity (*m_x_*) was calculated as

(2) $$m_{x}=\overset{m}{\underset{j=1}{\sum }}s_{xj}f_{xj}/\overset{m}{\underset{j=1}{\sum }}s_{xj}$$

The net reproductive rate, which is defined as the total number of offspring that an individual can produce during its lifetime, is calculated as

(3) $$R_{0}=\overset{\infty }{\underset{x=0}{\sum }}l_{x}m_{x}$$

The intrinsic rate of increase was calculated using the Lotka-Euler equation with age indexed from 0 as

(4) $$\overset{\infty }{\underset{x=0}{\sum }}e^{-r\left(x+1\right)}l_{x}m_{x}=1$$

The mean generation time represents the period that a population requires to increase to *R*_0_-fold of its size as time approaches infinity and the population settles down to a stable age-stage distribution. Mean generation time is calculated as

(5) $$T=\frac{\mathrm{In}R_{0}}{r}$$

Age-stage-specific life expectancy (*e_xy_*) (i.e., the time that an individual of age *x* and stage *y* is expected to live) was calculated using the method described previously by Chi and Su [17] as

(6) $$e_{xy}=\overset{n}{\underset{i=x}{\sum }}\overset{m}{\underset{j=y}{\sum }}s_{ij}^{\prime }$$

where *s′_ij_* is the probability that an individual of age *x* and stage *y* will survive to age *i* and stage *j*. In the age-stage, two-sex life table, it is calculated as

(7) $$v_{xy}=\frac{e^{-r\left(x+1\right)}}{s_{xy}}\overset{n}{\underset{i=x}{\sum }}e^{-r\left(i+1\right)}\overset{m}{\underset{j=y}{\sum }}s_{ij}^{\prime }f_{ij}$$

### 2.5. Population Projection of S. frugiperda

The computer program TIMING-MSChart [23] was used to project the population growth of *S. frugiperda* on different plant species for over 4 months.

### 2.6. Statistical Analysis

The means and standard errors of developmental time, prepupa (from the last-stage larva that is often quiescent to before the ecdysis to pupa), preadult (from egg to adult emergence), longevity, fecundity, and population parameters were calculated using the bootstrap method with 100,000 replicates, and the differences among treatments were measured and compared using the Tukey-Kramer procedure [24]. The effects of different plant species on pupal weight were subjected to one-way analysis of variance (ANOVA), and an independent samples t-test was applied for the male and female pupae from the same plant species data to analyze the significance at *p* < 0.05. The experimental data were analyzed using the package IBM SPSS Statistics 20 (SPSS, Inc., Chicago, IL, USA).

## 3. Results

### 3.1. Life History of S. frugiperda

When reared on Chinese cabbage, only three *S. frugiperda* pupae were observed to develop into adults; therefore, the durations of the pupa stage, preadult (from egg to adult emergence) stage and adult longevity could not be compared with those of *S. frugiperda* reared on the other five plant species (Table 1). The time for embryo development was calculated to be the longest when reared on soybean (2.95 days), followed by maize (2.49 days), and it was the shortest when reared on other plant species (2.00 days). The pupal stage varied significantly from 10.13 days on cotton to 8.90 days on maize (*F* = 29.15; *df* = 4, 342; *p* < 0.001). The longest preadult duration periods were found to be associated with tomato (38.06 days) and Chinese cabbage (37.33 days); and the shortest duration periods were associated with maize (24.67 days) and wheat (25.18 days). The longevity of female adults was longer than males, except when reared on soybean (Table 1).

**Figure 1:**
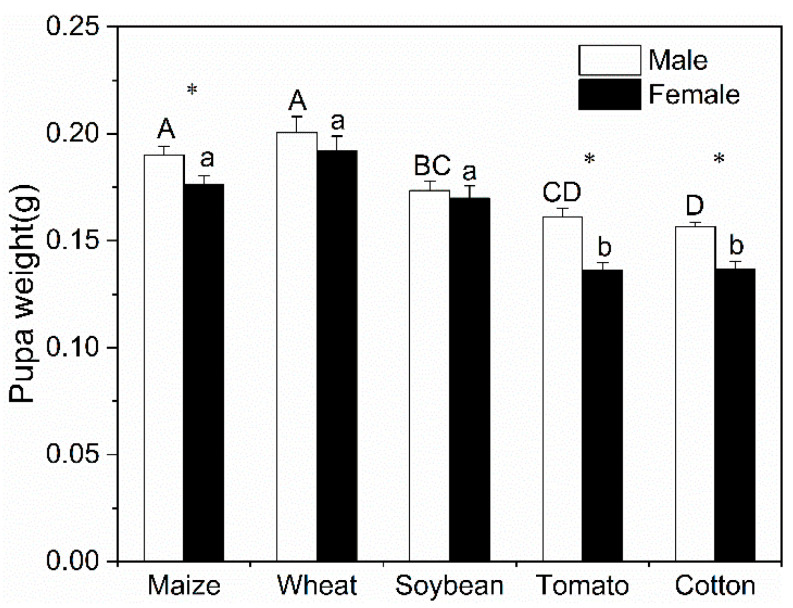
Pupa weight of *S. frugiperda* fed on five plant species. Different uppercase and lowercase letters indicate significant differences among different plant species (*p* < 0.05), respectively. * indicates significant differences between females and males on the same plant species (*p* < 0.05).

When *S. frugiperda* was reared on soybean, tomato and cotton, 14.9%, 9.7% and 57.7% of six instar larvae pupated, respectively, but 36.6%, 58.3% and 32.7% of the six instar larvae continued to develop into the seventh instar stage and pupated, respectively. Female and male pupae have showed significant variation in weights when reared on different plant species (Figure 1). Male pupae from wheat and maize were remarkably heavier than the ones from cotton and tomato (*F* = 18.87; *df* = 4, 203; *p* < 0.001). Similarly, female pupae from maize, wheat and soybeans were significantly heavier than those from tomato and cotton (*F* = 32.12; *df* = 4, 117; *p* < 0.001). The male pupae were notably heavier compared to the female pupae when reared on maize (*t* = −2.378, *df* = 79, *p* = 0.020), tomato (*t* = 4.446, *df* = 82, *p* < 0.001) and cotton plants (*t* = 5.183, *df* = 99, *p* < 0.001).

The adult preoviposition period (APOP), total preoviposition period (TPOP), female proportions (*N_f_*/*N*), male proportions (*N_m_*/*N*), oviposition days, fecundities and egg hatching rates of the *S. frugiperda* cohorts reared on six plant species are shown in Table 2. The shortest APOP was 2.89 days on maize, and the longest was 4.72 days on cotton. Similarly, the shortest TPOP was 26.85 days on maize, and the longest was 41.33 days on tomato. The proportions of females and males greatly varied from 0.447 to 0.188 and 0.528 to 0.198 on different plant species. The longest oviposition day was 8.11 days on cotton, and the shortest was 4.70 days on tomatoes. Fecundity varied significantly from 1275.56 eggs on maize to 586.17 eggs on tomato. The egg hatching rates notably varied from 95.94% to 83.53% on different plant species.

### 3.2. Population Parameters of S. frugiperda

The values of net reproductive rate (*R*_0_ = 406.37), intrinsic rate of increase (*r* = 0.2056) and finite rate of increase (*λ* = 1.2283) on maize were significantly higher than those on other plant species (Table 3). The mean generation time (*T*) was found to be highest on tomato (42.96 days) and cotton (42.55 days) and lowest on maize (29.21 days) and wheat (29.58 days). Due to the low fecundity and hatch rates, the *R*_0_, *r* and *λ* were lowest on Chinese cabbage.

### 3.3. Life Table Analysis

**Figure 2:**
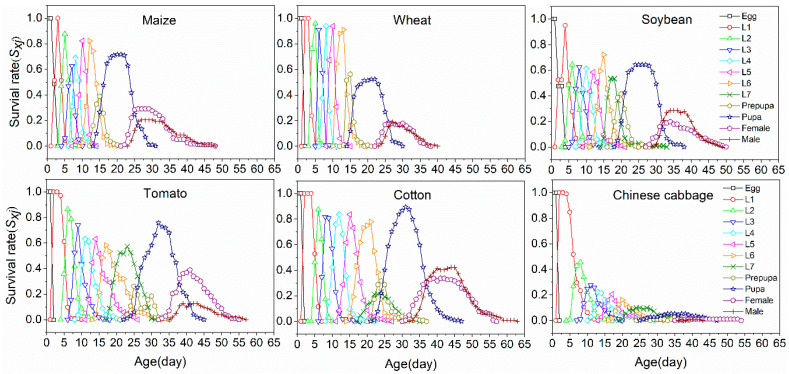
Age-stage specific survival rate (*s_x_*_j_) of *S. frugiperda* fed on six plant species.

The age-stage specific survival rate (*s_xj_*) represented the probability that a *S. frugiperda* egg will survive to age *x* and stage *j* on six plant species (Figure 2). Due to variable developmental rates among individuals, significant overlaps between stages were observed among the six host plants of *S. frugiperda*. The highest larval survival rate was observed on cotton, and 90.1% of eggs normally survived to the adult stage, followed by tomato (62.1%), maize (54.0%), soybean (51.5%) and wheat (38.6%), while the lowest survival rate (2.7%) was observed on Chinese cabbage. Furthermore, female adults emerged earlier than male adults on each plant species.

**Figure 3:**
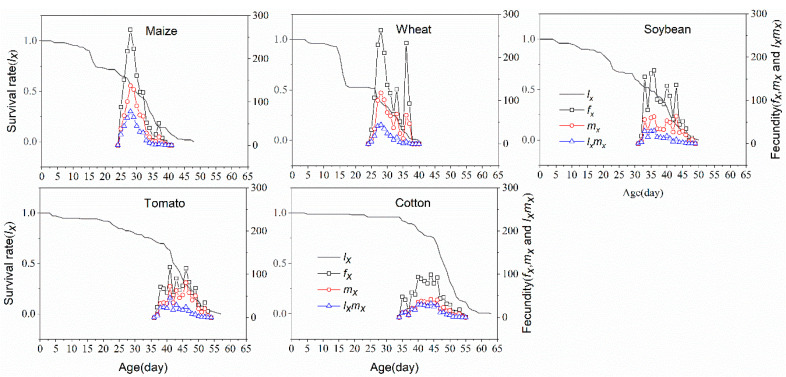
Age-specific survival rate (*l_x_*), female age-stage specific fecundities (*f_x_*), fecundity (*m_x_*) and net maternity (*l_x_m_x_*) of *S. frugiperda* fed on five plant species.

The age-specific survival rate (*l_x_*), the female age-stage specific fecundity (*f_x_*), the age-specific fecundity (*m_x_*) and the age-specific net maternity value (*l_x_m_x_*) of *S. frugiperda* cohorts reared on five plant species are illustrated in Figure 3. The *l_x_* curve slowly decreased from 100% to 74.3% on maize and from 100% to 54.5% on wheat in the first 14 days, respectively. After 14 days, the survival rate has quickly dropped to 0%. On soybean, tomato and cotton plants, the *l_x_* curves showed a similar trend of steady decline during early development stages and then a rapid decline toward the end of development. The *m_x_* and *f_x_* curves had been observed to have similar trends on all plant species. On maize and wheat, the *f_x_* and *m_x_* curves reached reproductive peaks at 28 days of age, with the highest fecundity being 267 and 263 hatched eggs, respectively. However, the lowest fecundity was on cotton, with 98 eggs at 44 days. Moreover, the *f_x_* curve on maize only had one peak, whereas there were two or more peaks on other plant species curves, suggesting that the oviposition periods of *S. frugiperda* on these plants were not concentrated and there were relatively significant differences among individuals.

**Figure 4:**
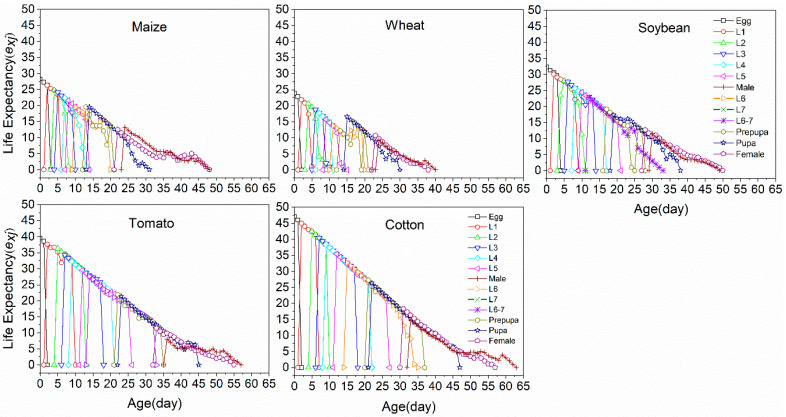
Age-stage specific life expectancy (*e_xj_*) of *S. frugiperda* fed on five host crops.

The age-specific life expectancy (*e_xj_*) represented the length of time that a *S. frugiperda* individual of age *x* and stage *j* is expected to survive after age *x* (Figure 4). The average life expectancy values of *S. frugiperda* individuals that were reared on maize, wheat, soybean, tomato and cotton were 28.30, 23.84, 32.3, 39.57 and 47.01 days, respectively.

**Figure 5:**
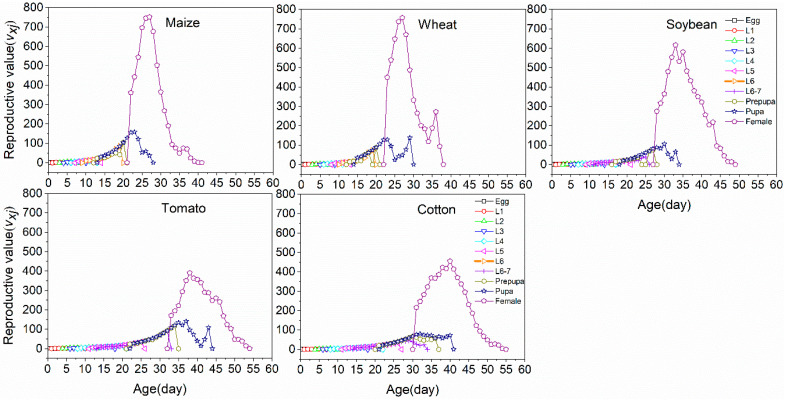
Age-stage specific reproductive value (*v_xj_*) of *S. frugiperda* fed on five host crops.

The age-stage specific reproductive values (*v_xj_*) of *S. frugiperda* represented the contribution of an individual at age *x* and stage *j* to the future population (Figure 5). The reproductive value increased significantly when *S. frugiperda* began laying eggs. Increases in reproductive values occurred at 23–29, 23–29, 30–38, 34–44 and 33–45 days on maize, wheat, soybean, tomato and cotton, respectively. On maize and wheat, the reproductive peaks occurred much earlier at 27 days of age, which reached peaks of 751.3 and 756.1, respectively. In contrast, the peaks of reproductive values that occurred later at 38 and 40 days and were as high as 390 and 454 eggs on tomato and cotton, respectively.

### 3.4. Population Projection of S. frugiperda

**Figure 6:**
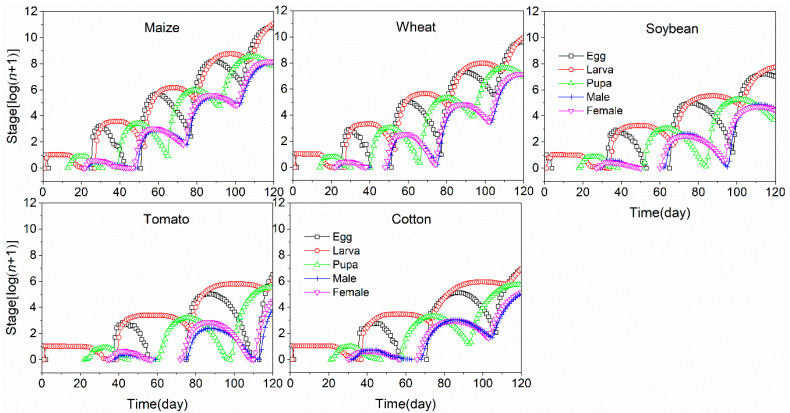
Projection of population growth potential of *S. frugiperda* fed on five host crops for duration of 120 days.

The growth capacities of *S. frugiperda* were projected using life table data to demonstrate the increase in the pest population and the stage structure during the process of population growth on different plant species (Figure 6). As the age-stage, two-sex life table can integrate stage differentiation and include the influence of gender, the derived population prediction has the ability to accurately describe the details of stage structure. The population growths were faster when *S. frugiperda* was reared on maize and wheat, whereas the population grew slowly on tomato and cotton. Furthermore, in the prediction period of 120 days, *S. frugiperda* is expected to occur for five generations on maize and wheat and for three generations when fed on tomato and cotton.

**Figure 7:**
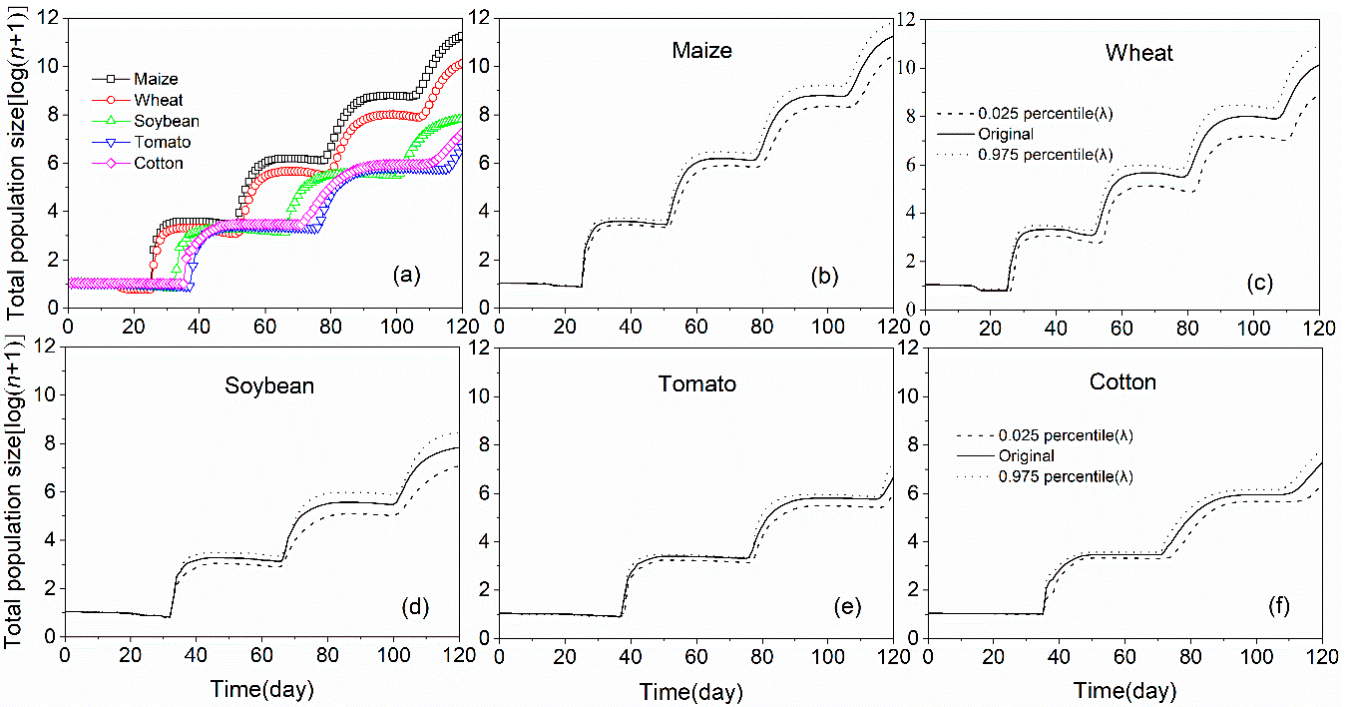
Projection of population growth potential of *S. frugiperda* fed on five plant species (**a**); the uncertainty of population projection of *S. frugiperda* fed on five plant species (**b**–**f**).

In the prediction period of 120 days, the total population size was the highest on maize (11.25), followed by wheat (10.13) and the lowest on tomato (6.69) on a log scale, respectively (Figure 7a). Alternatively, the population growths of *S. frugiperda* on five plant species were highly uncertain; this could be attributed to differences in developmental speed, viability and fertility among individuals (Figure 7b–f). The variability of the projected population growth, which was simulated using the 2.5th and 97.5th percentiles of the *λ* for bootstrap with 100,000 replications, which indicated that there was a high degree of uncertainty of *S. frugiperda* population on wheat at 120 days.

## 4. Discussion

Plant species have a significant effect on the development, survival and reproduction of herbivorous insects. In general, shorter developmental times and higher reproduction rates on a certain plant species represented higher suitability of the plant [16,25]. Although more than 350 host plants have been recorded, their influence on the life history of *S. frugiperda* has not been thoroughly examined [2]. The findings obtained in this study suggest that the development, survivorship, reproduction and population growth of *S. frugiperda* cohorts can significantly vary depending on the plant species. In this present study, we found that the six plant species support the development of *S. frugiperda*, but the biological characteristics varied significantly across the host plants. *Spodoptera frugiperda* experienced the shortest larval developmental duration and TPOP, the longest adult longevity, the greatest pupa weight and the highest fecundity when feeding on maize and wheat compared to other plant species. These results were consistent with previous reports on cotton [26,27]. More interestingly, our results found that when *S. frugiperda* fed on Chinese cabbage, only 5.3% of the initial larvae developed into adults and laid eggs, but the eggs did not hatch, suggesting that Chinese cabbage could be an unsuitable host plant for *S. frugiperda* and that the pest could not establish a population on it. However, as a migratory pest, *S. frugiperda* might use Chinese cabbage as a supplementary food; *S. frugiperda* larvae were voracious, so the pest might still cause economic losses in Chinese cabbage. In fact, it has been reported that *S. frugiperda* larvae infested Chinese cabbage in the field [28]. Additionally, we speculated that the high mortality rate of *S. frugiperda* feeding on Chinese cabbage might be related to poor nutrition or some other components (e.g., large amounts of anthocyanins and myrosinase) in Chinese cabbage.

Plant species affect not only the growth and development of phytophagous insects but also the number of larval instars and pupa weights. In this study, we found that *S. frugiperda* larvae had six instars when feeding on maize or wheat; however, when *S. frugiperda* fed on soybean, tomato and cotton, some larvae pupated at the sixth instar stage and some at the seventh instar stage. Pencoe and Martin [29,30] found that *S. frugiperda* had seven, eight, or nine instars when reared on some wild grasses. However, this is the first report to reveal that there were seven instars of *S. frugiperda* larvae when fed on cash crops. Various factors (e.g., temperature, photoperiod, food quality and quantity, humidity, rearing density, inheritance and sex) have been shown to influence the number of insect instars [31]. The increase in insect instar number may be a double-edged sword. On the one hand, the older larvae of Noctuidae (including *S. frugiperda*) were considered gluttonous and eating more plant food could cause more economic losses to the plants; on the other hand, the prolongation of the larval stage might increase the likelihood of being attacked by natural enemies, which might be detrimental to population development. Whether the ratio of the sixth and seventh instar larvae can be determined by *S. frugiperda* according to the change in environmental conditions requires further study. Our results showed that *S. frugiperda* larvae fed on maize and wheat had a shorter larval duration and were heavier pupa. The results were consistent with the findings of Ba et al. [26] and Wu et al. [32]. Furthermore, in the current study, we found that there was a positive relationship between weight of female pupa and fecundity of *S. frugiperda*, i.e., when fall armyworm fed on maize, wheat or soybeans, its pupal weight and fecundity were significantly higher than that on other plants. The results indicated that maize, wheat or soybeans were the most suitable host plants for the growth and development of *S. frugiperda*, which suggest that this pest will cause serious economic losses to these cash crops. More interestingly, the present results found that female *S. frugiperda* pupae emerged 1–3 days earlier than male pupae. We speculate that this phenomenon might be attributed to the migratory characteristics of *S. frugiperda*. Females that emerge early are more likely to disperse because they need to find food and locate suitable oviposition sites. Similar to our results, earlier female emergence has been reported in migratory insects such as *Pseudaletia unipuncta* (Haworth) (Lepidoptera: Noctuidae) [33], *Helicoverpa armigera* (Hübner) (Lepidoptera: Noctuidae) [34] and *Cnaphalocrocis medinalis* (Guenée) (Lepidoptera: Pyralidae) [35]. Furthermore, migratory female insects possess a greater migration capacity than males, which ensures the rapid expansion of migratory populations.

The parameters of life tables often vary with temperature, chemical pesticides and host species [36,37,38,39,40,41]. The *r* summarizes the physiological qualities of an animal relative to its capacity of increase and is often used to compare the fitness of populations across diverse climatic and food-related conditions [25,42]. Therefore, the stronger the adaptability of an insect population, the higher the *r* value. The *r* values for *S. frugiperda* were reported as 0.1678 and 0.1526 day^−1^ on maize and wheat [26], which were significantly lower than our results. This could be attributed to the enhanced ability of *S. frugiperda* to adapt to a complex environment. Besides this, genetic differences among the pest strains used in those studies might be a reason. In this present study, both the APOP and TPOP of *S. frugiperda* on maize and wheat were found the shortest, resulting in the highest *r* values of increase. The overlap in the *s_xj_* curves indicated the variable developmental rates in stage differentiation among *S. frugiperda* individuals. These overlaps were present in most of the life history data. Similarly, the differences in life expectancy and reproductive value among individuals of the same age but different stages can also be observed in the *e_xj_* and *v_xj_* curves. The *e_xj_* is calculated using the *s_xj_* without assuming that the population reaches a stable age-stage distribution. Therefore, it can be used to predict the survival of a population at that condition. It is essential to accurately predict the development trend of the pest population to formulate pest management strategies. A computer projection of pest population growth using life table data is an indispensable tool in pest management and decision-making [18]. At present, the management of *S. frugiperda* in China is mainly based on monitoring *S. frugiperda* male adults using pheromone traps, and then an emergent chemical control is recommended. However, our forecast results showed that the dynamics of male *S. frugiperda* adults were different from female adults. Therefore, we suggest that the current pheromone-monitoring program be reassessed and adjusted to more accurately predict the occurrence of *S. frugiperda*.

## 5. Conclusions

In summary, this present study represents the first comprehensive report on the life history data of *S. frugiperda* on six cash crop species using the age-stage, two-sex life table. This study indicated that *S. frugiperda* cohorts that were reared on maize and wheat had shorter preadult developmental durations, higher preadult survival, shorter APOP and TPOP, greater pupal weights and higher fecundity compared to the other four plants. The combined effects of these factors resulted in *S. frugiperda* having a greater *R*_0_, *r* and *λ* when reared on maize and wheat compared with those reared on soybean, tomato, cotton and Chinese cabbage. These demographic parameters explain the strong environmental adaptability of *S. frugiperda*, which is responsible for the serious damage to maize in China and the rest of the world. Furthermore, according to the present study, we suggest that *S. frugiperda* could also cause great economic losses to wheat, soybean, tomato, cotton or Chinese cabbage, which should attract the attention of agricultural management departments. Our findings provide useful information in predicting population dynamics and understanding the potential damage that could be incurred by *S. frugiperda* infestation.

## Figures and Tables

**Table 1:** Developmental time and longevity (M ± SE) of different life stages of *S. frugiperda* fed on six plant species.

Stage (Days)	N	Maize	N	Wheat	N	Soybean	N	Tomato	N	Cotton	N	Chinese Cabbage
Egg	113	2.49 ± 0.05 b	101	2.00 ± 0 c	101	2.95 ± 0.10 a	103	2.00 ± 0 c	104	2.00 ± 0 c	112	2.0 ± 0.04 b
First instar	111	2.07 ± 0.02 e	97	2.21 ± 0.04 d	99	2.84 ± 0.06 c	98	3.74 ± 0.08 b	103	3.65 ± 0.07 b	57	4.77 ± 0.15 a
Second instar	110	1.93 ± 0.03 e	97	1.87 ± 0.04 e	96	2.05 ± 0.03 d	98	2.89 ± 0.09 b	103	2.20 ± 0.04 c	48	3.79 ± 0.15 a
Third instar	109	1.49 ± 0.05 e	96	1.57 ± 0.05 e	95	2.03 ± 0.02 d	98	2.60 ± 0.09 c	103	2.94 ± 0.10 b	39	3.46 ± 0.19 a
Fourth instar	106	1.74 ± 0.05 d	96	1.69 ± 0.05 d	92	2.07 ± 0.04 c	97	2.89 ± 0.08 b	103	3.47 ± 0.06 a	34	3.32 ± 0.17 a
Fifth instar	105	2.08 ± 0.04 d	95	2.08 ± 0.03 d	91	2.29 ± 0.05 c	96	3.42 ± 0.17 b	102	3.81 ± 0.10 a	24	3.17 ± 0.16 b
Sixth instar	100	2.95 ± 0.10 d	89	3.33 ± 0.08 b	87	2.97 ± 0.08 c	94	4.29 ± 0.15 b	102	4.94 ± 0.13 a	17	3.88 ± 0.33 b
Seventh instar	-	-	-	-	37	3.59 ± 0.12 b	60	5.77 ± 0.17 a	34	5.44 ± 0.13 a	10	7.00 ± 0.78 a
Larvae	100	12.21 ± 0.11 f	89	12.66 ± 0.11 e	83	16.65 ± 0.16 d	87	24.74 ± 0.26 b	101	22.81 ± 0.27 c	10	29.10 ± 0.32 a
Prepupa	82	1.39 ± 0.06 cd	54	1.30 ± 0.07 d	76	1.83 ± 0.05 a	83	1.54 ± 0.05b c	99	1.59 ± 0.06 b	9	1.56 ± 0.22 abcd
Pupa	64	8.90 ± 0.10 b	42	8.97 ± 0.13 b	55	9.90 ± 0.13 a	73	9.96 ± 0.10 a	97	10.13 ± 0.09 a	3	10.33 ± 0.49
Preadult	64	24.67 ± 0.16 d	42	25.18 ± 0.23 d	55	31.50 ± 0.24 c	73	38.06 ± 0.30 a	97	36.64 ± 0.31 b	3	37.33 ± 0.62
Adult longevity												
Female	36	16.21 ± 0.11 a	19	13.01 ± 1.41 a	21	9.33 ± 1.84 b	40	13.04 ± 1.52 a	39	16.22 ± 1.89 a	1	18.0 ± 0
Male	25	9.09 ± 1.64 a	20	7.11 ± 1.53 b	31	9.21 ± 0.70 a	24	7.04 ± 0.64 b	55	9.13 ± 0.61 a	2	2.5 ± 0

Means in the same row followed by different letters are significantly different (*p* < 0.05).

**Table 2:** APOP, TPOP, female and male proportions, mean fecundity and egg hatching rate (M ± SE) of *S. frugiperda* fed on six plant species.

Stage	Maize	Wheat	Soybean	Tomato	Cotton	Chinese Cabbage
APOP (d)	2.89 ± 0.21 c	3.39 ± 0.34 bc	4.10 ± 0 a	4.08 ± 0.24 b	4.72 ± 0.44 a	4.0
TPOP (d)	26.85 ± 0.27 e	27.94 ± 0.48 d	34.65 ± 0.39 c	41.33 ± 0.39 a	39.83 ± 0.51 b	40.0
Female proportion (*N_f_*/*N*)	0.318 ± 0.044 ab	0.188 ± 0.039 c	0.208 ± 0.040 bc	0.447 ± 0.049 a	0.375 ± 0.047 a	-
Male proportion (*N_m_*/*N*)	0.221 ± 0.039 b	0.198 ± 0.039 b	0.307 ± 0.046 b	0.233 ± 0.042 b	0.528 ± 0.049 a	-
Oviposition days (d)	7.22 ± 0.55 ab	4.89 ± 0.64 c	5.65 ± 0.69 bc	4.70 ± 0.39 c	8.11 ± 0.39 a	12
Fecundity(*F*) (eggs)	1275.56 ± 155.29 a	1180.84 ± 179.15 ab	963.86 ± 144.89 ab	586.17 ± 69.78 c	803.51 ± 75.33 b	112.90
Hatching rate	0.9496 ± 0.1438 a	0.8938 ± 0.0408 ab	0.9594 ± 0.0091 a	0.9248 ± 0.0238 ab	0.8353 ± 0.0280 b	0

Means in the same row followed by different letters are significantly different (*p* < 0.05).

**Table 3:** Population parameters of *S. frugiperda* fed on six plant species.

Plant Species	*R*_0_	*r* (Day^−1^)	*λ* (Day^−1^)	*T* (Day)
Maize	406.37 ± 74.43 a	0.2056 ± 0.0072 a	1.2283 ± 0.0088 a	29.21 ± 0.32 c
Wheat	222.14 ± 56.53 b	0.1827 ± 0.0101 a	1.2004 ± 0.0120 a	29.58 ± 0.40 c
Soybean	200.41 ± 48.73 b	0.1418 ± 0.0075 b	1.1524 ± 0.0086 b	37.37 ± 0.63 b
Tomato	261.79 ± 42.18 ab	0.1296 ± 0.0044 b	1.1384 ± 0.0050 b	42.96 ± 0.52 a
Cotton	301.32 ± 47.40 ab	0.1342 ± 0.0046 b	1.1436 ± 0.0053 b	42.55 ± 0.65 a
Chinese cabbage	8.0	0.0444	1.0454	46.81

Means in the same column followed by different letters are significantly different (*p* < 0.05).
